# Correlation of P-wave velocity with mechanical and physical properties of limestone with statistical analysis

**DOI:** 10.1038/s41598-021-03524-0

**Published:** 2021-12-16

**Authors:** Hasan Arman

**Affiliations:** College of Science, Geosciences Department, United Arab Emirates University, P.O. Box: 15551, Al Ain, UAE

**Keywords:** Environmental sciences, Materials science

## Abstract

This study aims to investigate the correlation between the P-wave velocity (V_p_) and the mechanical and the physical properties of the limestone; V_p_ tests were conducted on over 320 limestone samples. Moreover, the effects of the mineralogical, textural, and chemical composition of limestone were also studied through thin sections, scanning electron microscopy (SEM), X-ray diffraction (XRD), and X-ray fluorescence (XRF). The relationships between the V_p_ and the uniaxial compressive strength (UCS), point load index (PLI(I_s(50)_), 2nd cycle of slake durability index (I_d2_), natural unit weight (γ_n_), specific gravity (G_s(c)_), water absorption by weight (WA), and porosity (n) were estimated using representative empirical equations. The empirical equations were validated by Student’s* t* test that has indicated the existence of strong relationships between the mechanical and physical properties of the intact limestone with V_p_; the calculated t-values were higher than the t-critical value. Furthermore, the results of previously available studies were compared with the results of this study in terms of the generated equations for V_p_ values and the slope of a 1:1 line, which was used to appraise the predicted and measured values. This study demonstrates that the UCS, PLI(I_s(50)_), I_d2_, γ_n_, G_s(c)_, WA, and n values of an intact limestone can be predicted by using V_p_, which is fast, easy, economical and nondestructive test.

## Introduction

Seismic techniques are well understood, nondestructive, and low-cost methods that can be easily performed in the laboratory and in-situ. These techniques are widely used in civil, geological, mining, and rock engineering applications to characterize the dynamic properties of rocks. The seismic properties of rocks are influenced by numerous factors, such as the rock type, texture, mineralogical composition, grain shape and size, density, porewater, porosity, and water content^1–6^.

Characterization of rock materials in the field or laboratory requires the measurement of the mechanical and physical properties of rocks, which are critical tasks in many geotechnical and rock engineering projects. Moreover, such experimental studies are extremely expensive, tedious, and time consuming. Reliable modeling of empirical relationships among the mechanical and physical properties of rock materials can eliminate the need for such an expensive and tedious work and facilitate the estimation of the necessary critical design parameters.

Numerous researchers have already derived possible relationships between P-wave velocity (V_p_) and the mechanical and physical features of limestone using statistical methods like regression analyses (Table 1). Tugrul and Zarif^1^ examined the relationships between total porosity (n_t_), uniaxial compressive strength (UCS), and V_p_ using linear empirical equations and found strong negative and positive relationships among the correlated parameters. Using simple linear relations, Yasar and Erdogan^2^ reported strong relationships among UCS, modulus of elasticity (E), density (ρ), and V_p_. Kahraman and Yeken^3^ conducted various tests, for instance, absorption of water by weight (WA), ρ, porosity (n), and V_p_, on 14 different carbonate rocks and reported strong negative and positive correlations between V_p_ and all studied physical parameters. Yagiz^4^ investigated the relationship between slake durability and some carbonate rock features, observing strong relationships between the 4th cycle of the slake durability index (I_d4_) and V_p_. Yagiz^7^ assessed the geotechnical properties of carbonate rocks using V_p_ tests and found high to low positive and negative correlation coefficients in the relationships among UCS, E, effective porosity (n′), WA, Schmidt rebound value (SHV), 2nd cycle of the slake durability index (I_d2_), dry density (ρ_d_), saturated density (ρ_s_), and V_p_ using statistical analyses, including Student’s *t* test. Arman et al.^5^ reported moderate correlations among indirect tensile strength (ITS), point load index (PLI), and V_p_. Using 45 limestone core specimens, Najibi et al.^8^ revealed relatively powerful relationships between E and UCS with V_p_. Stan-Kieczek^9^ studied the elastic properties of carbonate rocks via laboratory and in-situ tests and reported a strong relationship between E and V_p_. Pappalardo et al.^10^ performed a detailed laboratory characterization of the limestones of heritage Baroque monuments and discovered strong positive and negative correlations between limestone V_p_ and E, UCS, and n. Jamshidi et al.^11^ developed empirical equations to estimate the mechanical properties of travertine building stones from V_p_ and Schmidt hardness. Jamshidi et al.^12^ examined the effects of n and ρ on the relationship between UCS and V_p_ and noted a strong correlation between V_p_ and UCS. Ferriodooni and Khajevad^13^ investigated the relationships between engineering properties, slake durability index (SDI) of some travertine samples under the wetting–drying cycle, and observed a strong relationship between I_d2_ and V_p_. Using an indirect method of comparative evaluation to approximate the compressive strength of limestone, Ali et al.^14^ reported a strong correlation between V_p_ and UCS. Kurtulus et al.^15^ estimated the UCS using SHV and V_p,_ which proved to be strongly correlated. Wen et al.^6^ examined the correlation between geomechanics parameters and UCS and V_p_ on 40 dolomitic limestone specimens and reported significant correlations among the parameters of E, UCS, ρ, and Poisson’s ratio (ν).

**Table 1:** Data of the correlation between some mechanical and physical properties of either carbonate rocks or limestone from published literature and this study.

Researchers	Equations	R	Rock type
Tugrul and Zarif 2000^1^	*n*_*t*_ = *−0.62V*_*p*_ + *5.37*	− 0.78	Limestone
	*UCS* = *16.73V*_*p*_ + *21.25*	0.94	
Yasar and Erdogan 2004^2^	*V*_*p*_ = *0.0317UCS* + *2.0195*	0.89	Carbonates
	*V*_*p*_ = *0.0937E* + *1.7528*	0.93	
	*V*_*p*_ = *4.3183ρ –7.5071*	0.90	
Kahraman and Yeken 2008^3^	*WA* = *−2.248V*_*p*_ + *13.76*	− 0.95	Carbonates
	*ρ* = *0.213V*_*p*_ + *1.256*	0.91	
	*n* = *−4.733V*_*p*_ + *29.377*	− 0.94	
Yagiz 2011^4^	*I*_*d4*_ = *1.131V*_*p*_* – 93.26*	0.73	Carbonates
Yagiz 2011^7^	*E* = *20.1V*_*p*_*—53*	0.95	Carbonates
	*UCS* = *49.4V*_*p*_*—167*	0.89	
	*n′* = *−5.19V*_*p*_ + *27.1*	− 0.86	
	*WA* = *−2.23V*_*p*_ + *11.6*	− 0.85	
	*SHV* = *11.68V*_*p*_* – 6.64*	0.80	
	*I*_*d2*_ = *0.71V*_*p*_ + *95.7*	0.69	
	*ρ*_*d*_ = *0.19V*_*p*_ + *1.61*	0.58	
	*ρ*_*s*_ = *0.14V*_*p*_ + *1.88*	0.46	
Arman et al*.* 2014^5^	*PLI(I*_*s(50)*_*)* = *1.25V*_*p*_* – 5.4035*	0.61	Limestone
	*ITS* = *1.0051V*_*p*_* – 3.0393*	0.57	
Najibi et al*.* 2015^8^	*E* = *0.169V*_*p*_^3*.324*^	0.95	Limestone
	*UCS* = *3.67V*_*p*_^*2.14*^	0.90	
Stan-Kieczek 2016^9^	*E* = *74Ln(V*_*p*_*)*−*572*	0.86	Carbonates
Pappalardo et al*.* 2016^10^	*UCS* = *0.443e*^*(1.091Vp)*^	0.92	Limestone
	*UCS* = *10.57V*_*p*_*−19.9*	0.86	
	*E* = *2.784V*_*p*_*−5.434*	0.85	
	*n* = *0.9966e*^*(−0.333Vp)*^	− 0.97	
Jamshidi et al*.* 2016^11^	*UCS* = *101.1ln(V*_*p*_*)−802.8*	0.97	Travertine
	*ITS* = *8.44 ln(V*_*p*_*)−66.2*	0.96	
	*PLI* = *6.67 ln(V*_*p*_*)−51.9*	0.96	
Jamshidi et al*.* 2017^12^	*UCS* = *131.77ln(V*_*p*_*)−1048*	0.91	Limestone
Ferreidooni and Khajevad 2018^13^	*I*_*d2*_ = *0.0004V*_*p*_ + *96.34*	0.88	Travertine
Ali et al*.* 2018^14^	*UCS* = *0.045V*_*p*_ + *24.348*	0.81	Limestone
Kurtulus et al*.* 2018^15^	*V*_*p*_ = *166.5SHV–2698.1*	0.87	Limestone
	*V*_*p*_ = *268SHV–10,292*	0.92	
Wen et al*.* 2019^6^	*E* = *0.013V*_*p*_*–30.31*	0.91	Limestone
	*UCS* = *0.034V*_*p*_* –86.36*	0.89	
	*ρ* = 4.545*10^*−*5^*V*_*p*_ + *2.54*	0.82	
	*υ* = *0.52 – 91/(30.33* + *(V*_*p*_*)*^*0.69*^*)*	0.98	
This study	*UCS* = *20.395V*_*p*_* – 25.968*	0.67	Limestone
	*PLI(I*_*s(50)*_*)* = *0.8023V*_*p*_^*0.9448*^	0.71	
	*I*_*d2*_ = *3.6869ln(V*_*p*_*)* + *92.343*	0.72	
	*γ*_*n*_ = *18.328V*_*p*_^*0.1614*^	0.76	
	*G*_*s(C)*_ = *−0.342ln(V*_*p*_*)* + *2.5333*	− 0.76	
	*WA* = *−7.692ln(V*_*p*_*) –15.956*	− 0.87	
	*n* = *−18.47ln(V*_*p*_*)* + *38.8*	− 0.86	

*E:* Modulus of Elasticity (GPa), *UCS:* Uniaxial Compressive Strength (MPa)*, PLI(I*_*s(50)*_*):* Point Load Index value (for 50 mm in diameter size sample) (MPa), *ITS:* Indirect Tensile Strength (MPa), *SHV:* Schmidt Rebound Value (N), *V*_*p*_*:* P Wave Velocity (km/s or m/s), *γ*_*n*_ = natural unit weight (kN/m^3^). *WA* = Water Absorption (%)*, n, n*_*t*_*, n′* = Porosity, Total, Effective Porosity (%), *G*_*s(C)*_*:* Specific Gravity for core samples, *I*_*d2*_*, I*_*d4*_*:* Slake Durability Index, 2nd cycle, 4th cycle (I), *υ* = Poisson’s ratio, *ρ, ρ*_*d*_*, ρ*_*s*_ = Density, Dry Density, Saturated Density (g/cm^3^) and *R:* Regression Coefficient.

Herein, the relationship between the mechanical and physical properties of limestone and V_p_ were investigated. Further, the result of carbonate rocks or limestone obtained in previous studies were compared with those obtained in this study in terms of UCS, PLI(I_s(50)_), I_d2_, γ_n_, G_s(c)_, WA, and n. Moreover, the reliability of the empirical relationship was validated using Student’s* t* test and the predicted and measured cross-correlation values from V_p_.

## Study area and geological settings

**Figure 1:**
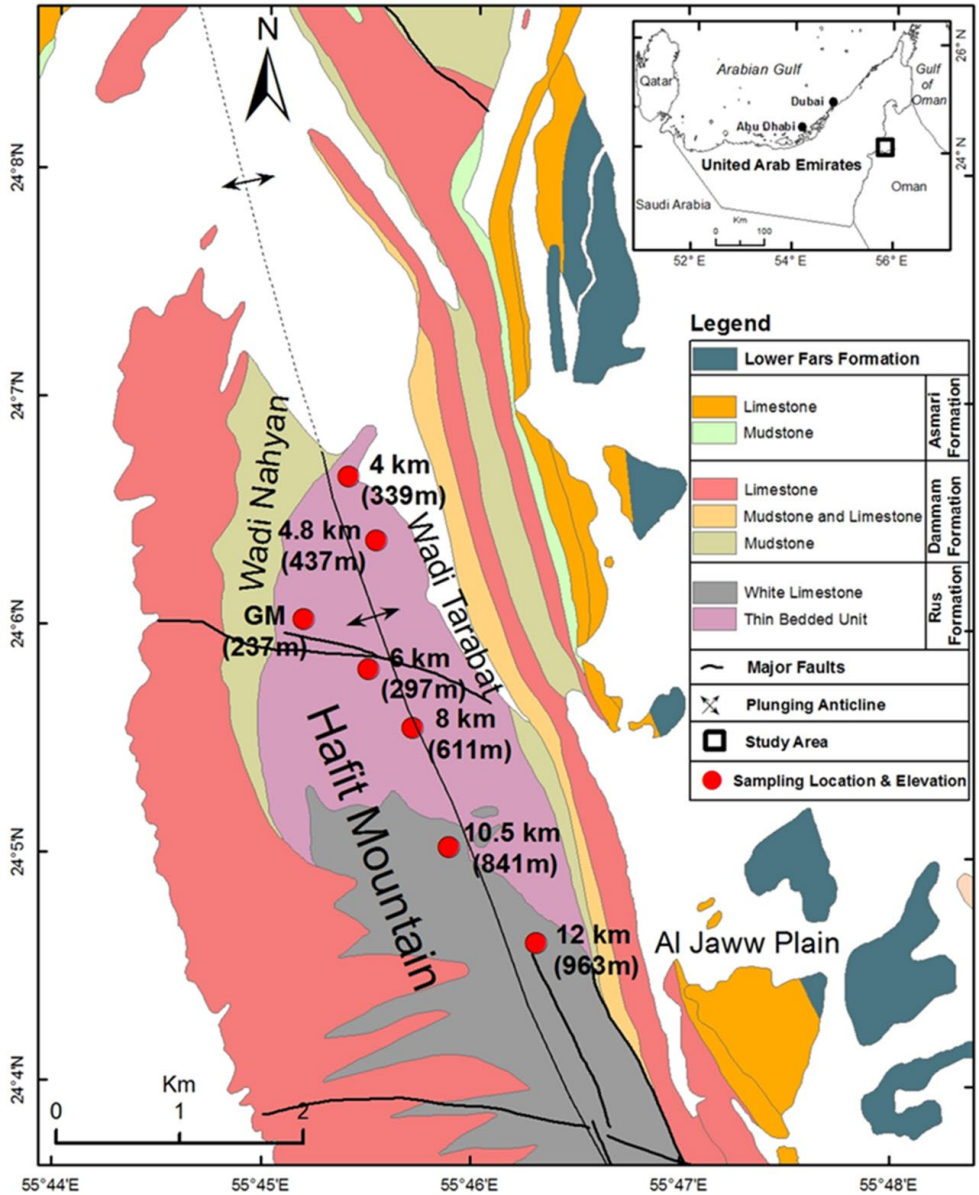
Location map of the sample sites with the geology of the Hafit Mountain (generated with ArcGIS 10.8^21^).

The study area, located along the Hafit Mountain, has been geologically well documented by numerous previous studies^16–20^ (Fig. 1). The carbonate rocks of the Hafit Mountain—an asymmetric and doubly plunging anticline—were dissected using numerous sets of faults. Tertiary carbonate rocks provide unique outcrops for three core rock units in the study area.

In the Hafit Mountain, the oldest rock unit is the Early Eocene Rus Formation, aged 55–49 Myr. It is thick bedded, massive, and generally appears grayish white in color. At some levels, brownish color chert nodules are dominated with dolomite layers. The Middle to Late Eocene Dammam Formation, aged 49–34 Myr, overlies the Rus Formation and exhibits some cavernous and fractured limestone layers, locally converting to chalky and dolomitic limestone with soft marl beds. Nummulitic limestone with marl beds is also available in different localized outcrops. The Early Oligocene Asmari Formation, aged 34–29 Myr, comprising mainly gypsiferous mudstone, nummulitic marly limestone, chalky and dolomitic limestone, and marl, is the youngest rock unit in the study area^22^.

The Early Miocene Lower Fars Formation, aged 23–16 Myr, comprises gypsite evaporates that are interbedded with friable marls and mudstones with gypsum veins, topped with a calcrete layer. The upper part comprises conglomeratic sandstone, rich in reworked chert and ophiolitic rock fragments^23^. The Miocene to Pliocene Barzaman Formation, aged 23–2 Myr, comprises a pebble–cobble conglomerate interbedded with sandstones and mudstones. The formation yields evidence for cycles of sedimentation from pluvial (wet) to arid, and the sediments of the arid intervals exhibit a white or pink color owing to dolomite alterations^24^.

## Rock sampling and laboratory studies

**Figure 2:**
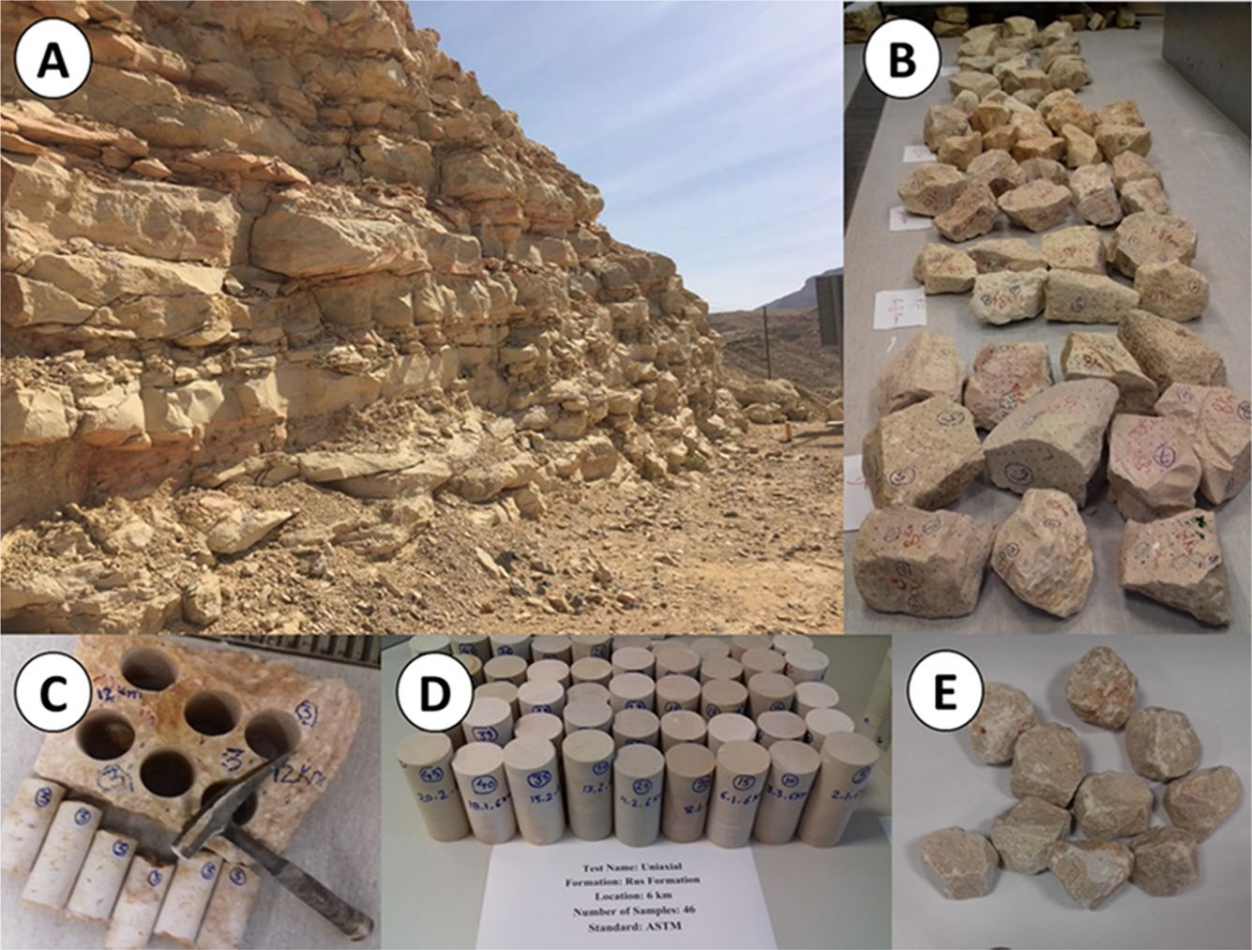
(**A**) General view of the Rus Formation and limestone outcrops, (**B**) a group of rock blocks collected from the field and used for coring and testing, (**C**) a cored rock block sample with core samples, (**D**) core samples prepared for the UCS test, and (**E**) test sample for the SDI test.

Over 100 limestone rock blocks were collected along the seven selected sampling locations (Figs. 1 and 2A, B). Each rock block was carefully inspected for laboratory testing and analysis. The rock blocks were appropriately represented and provided standard testing specimens without visible defects, such as alteration zones and fractures. According to the American Society for Testing and Materials^25^ standards, core samples were acquired from 94 selected rock blocks for physical and mechanical tests (Fig. 2C–E]). Table 2 lists the number of samples used for each test (from seven sampling locations) with their average (*x*) and standard deviation values. The tests were conducted on intact and natural rock samples. If the tests did not meet the suggested standards—owing to either core sample features or rock failing along the existing weakness plane—the results were not considered for examination.

**Table 2:** Number of samples used for each test and the range within one standard deviation of the average.

Sampling location (# Total samples)	V_p_ (km/s) x ± SD (# samples)	UCS (MPa) x ± SD (# samples)	PLI(I_s(50)_) (MPa) x ± SD (# samples)	I_d2_ (%) x ± SD (# samples)	γ_n_ (kN/m^3^) x ± SD (# samples)	G_s(C)_ x ± SD (# samples)	WA (%) x ± SD (# samples)	n (%) x ± SD (# samples)
1. (0 km) (212)	6.04 ± 0.94 (36)	86 ± 39 (16)	4 ± 1.6 (23)	98.35 ± 0.53 (13)	23.85 ± 1.10 (62)	1.96 ± 0.09 (36)	2.69 ± 1.44 (13)	6.67 ± 3.52 (13)
2. (4 km) (392)	4.93 ± 1.1 (71)	84 ± 38 (36)	4 ± 1.3 (37)	97.46 ± 1.34 (23)	24.20 ± 1.22 (108)	1.95 ± 0.12 (71)	3.37 ± 1.90 (23)	8.75 ± 4.45 (23)
3. (4.8 km) (102)	5.38 ± 0.88 (18)	85 ± 20 (9)	3 ± 0.6 (9)	97.97 ± 0.80 (7)	23.44 ± 0.89 (27)	2.01 ± 0.08 (18)	3.59 ± 1.23 (7)	9.49 ± 2.95 (7)
4. (6 km) (483)	5.13 ± 0.96 (92)	83 ± 41 (46)	4 ± 1.3 (46)	97.91 ± 0.95 (21)	23.70 ± 1.05 (144)	1.98 ± 0.09 (92)	3.36 ± 1.41 (21)	8.48 ± 3.42 (21)
5. (8 km) (227)	4.85 ± 1.74 (43)	86 ± 46 (21)	4 ± 1.4 (22)	95.78 ± 5.04 (11)	23.53 ± 1.96 (65)	2.03 ± 0.19 (43)	4.95 ± 4.28 (11)	12.37 ± 10.52 (11)
6. (10.5 km) (136)	6.94 ± 0.46 (23)	110 ± 31 (12)	9 ± 0.5 (11)	98.30 ± 0.35 (9)	25.04 ± 0.58 (40)	1.90 ± 0.06 (23)	1.27 ± 0.71 (9)	3.33 ± 1.87 (9)
7. (12 km) (201)	5.84 ± 0.35 (38)	68 ± 26 (19)	5 ± 0.9 (19)	98.37 ± 0.35 (10)	24.52 ± 0.31 (57)	1.92 ± 0.02 (38)	2.02 ± 0.57 (10)	5.41 ± 1.46 (10)

*x* = average and SD = standard deviation.

**Figure 3:**
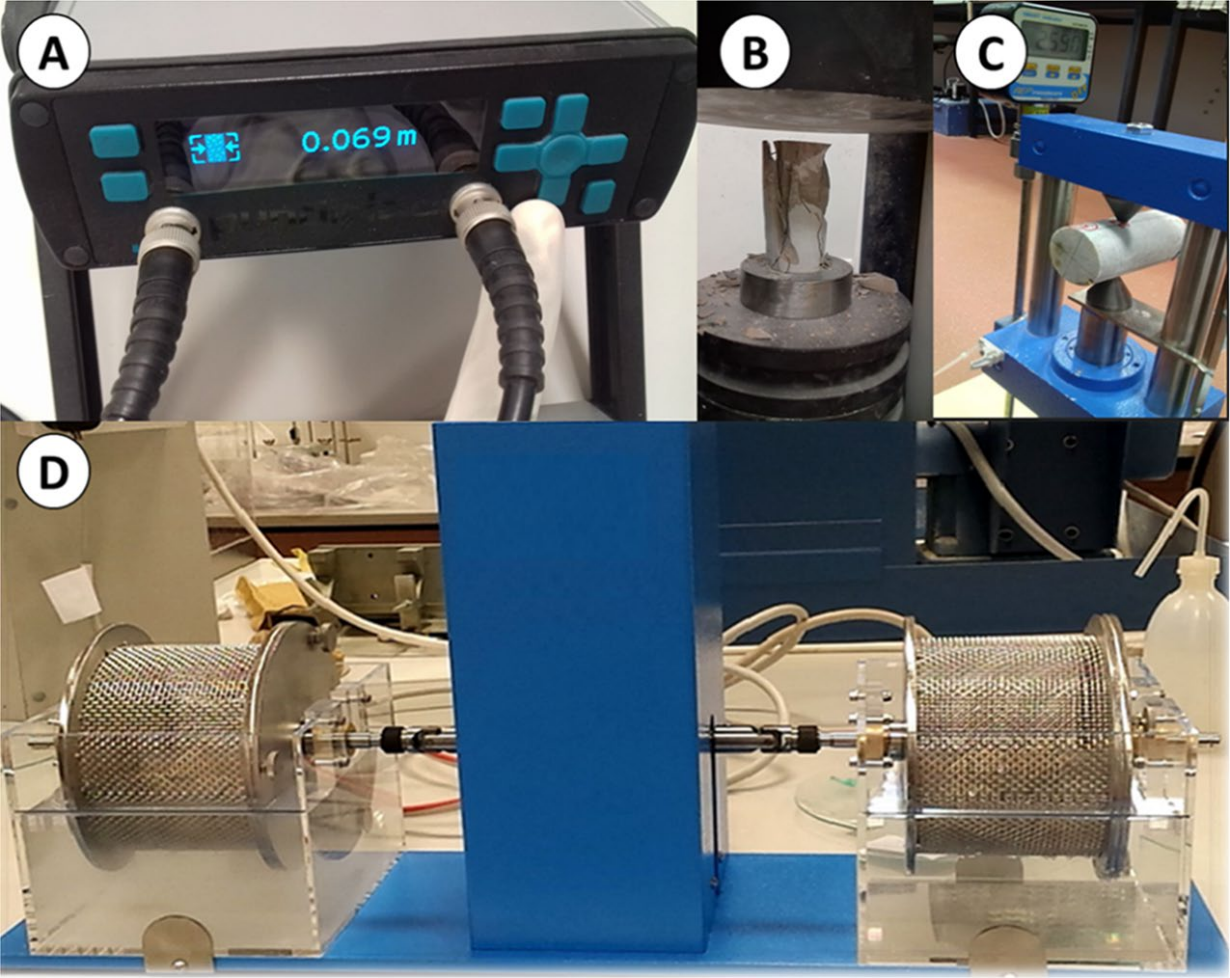
Laboratory equipment used for (**A**) V_p_ measurements, Pundit Lab, (**B**) UCS test, servo plus evolution press (**C**) PLI test, and (**D**) SDI test.

With reference to the ASTM^26^ standards, for the V_p_ test, a portable pulse generator unit control, Pundit Lab, and two transducers with 25.4-mm diameter and 250-kHz frequency were used to measure the V_p_ of the core samples (Fig. 3A). Further, 321 V_p_ tests were conducted on limestone core specimens. According to Anon^27^ classifications, the V_p_ of limestone indicates highly scattered ranges from 2.08 to 7.62 and can be classified as very low to very high.

The UCS, PLI, I_d2_, γ_n_, G_s(c)_, WA, and n values of the limestone samples were determined following the ASTM and ISRM standards. Table 2 lists the descriptive statistical distribution of the test results. The UCS tests were conducted on 159 NX-sized core samples, which were prepared based on the ASTM^28^ standard, with approximately 2:1 length to diameter ratio. Further, smooth sample end surfaces were prepared, and a 0.5–1-MPa constant loading rate was axially applied to the core specimens (Fig. 3B). The PLI test was conducted on 167 regular NX-sized core samples following the ASTM^29^ standard (Fig. 3C). If any sample failed either tests along existing cracks, weathered surfaces, or other weakness planes, the test results for such a sample was excluded. Moreover, 94 test samples were arranged for the SDI test from each rock block, and the SDI tests were conducted based on the ASTM^30^ standard (Fig. 3D). Based on a study by Franklin and Chandra^31^, I_d2_ was evaluated as a very high to extremely high SDI. The γ_n_ values of regular limestone core samples were calculated for approximately 500 test samples of UCS, PLI, and ITS test samples following the suggested method of the ISRM^32^. Based on the recommended methods of the ISRM^32^, 321 core samples were used to calculate the G_s(c)_. Further, the WA and n values were determined for each of the 94 limestone rock blocks using representative samples.

## Mineralogical and textural studies of rock units

**Figure 4:**
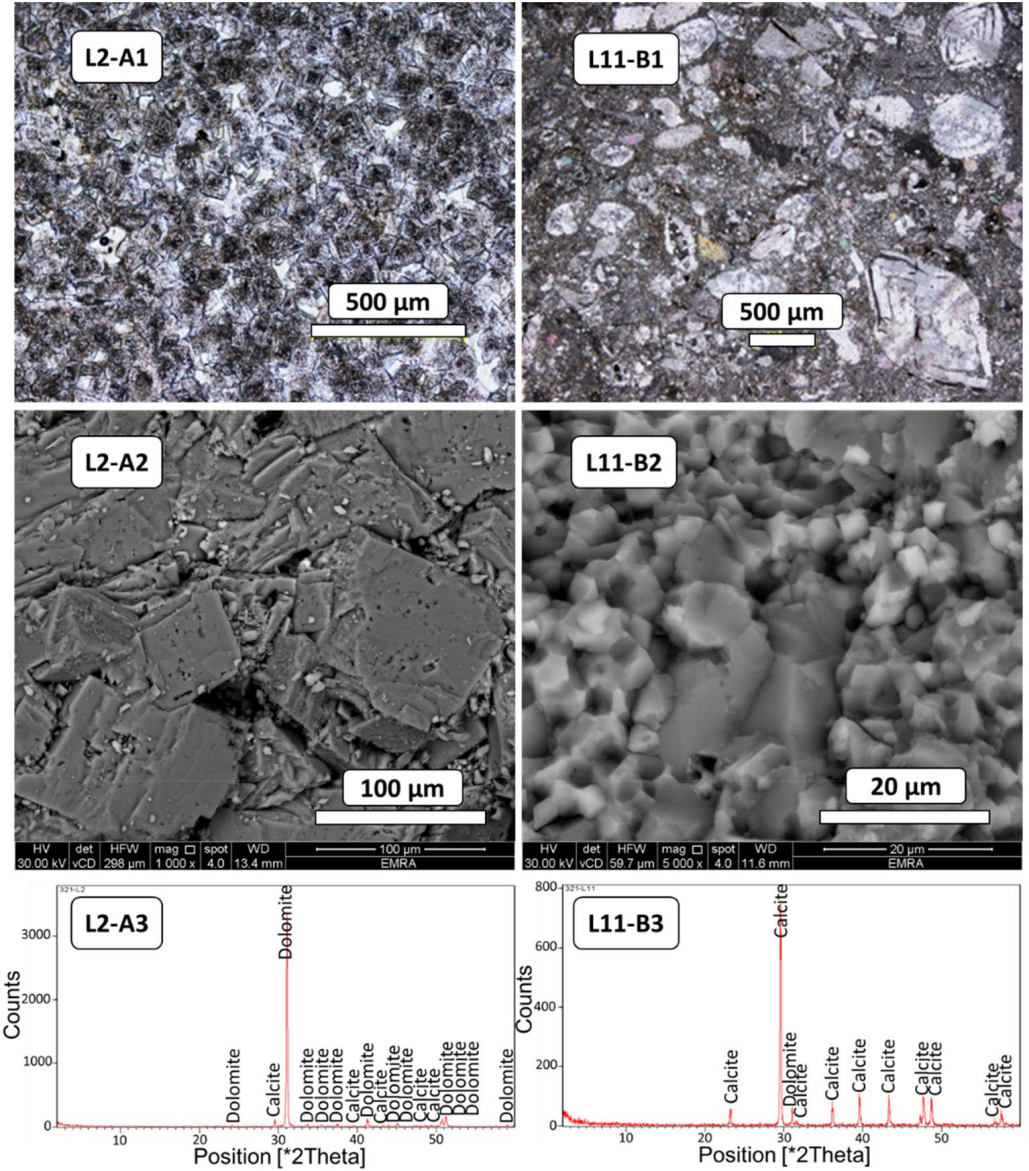
(L2–A1) Images of cross-polarized light showing relatively large dolomite crystals, (L2–A2) and (L2–A3) *showing texture and mineralogy of limestone:* calcite matrix and cement enlarged using SEM in (L2–A2), minerals identified using XRD in (L2–A3). (L11–B1) Images of cross-polarized light showing relatively large fossils embedded in a dominantly calcite matrix (L11–B2) and (L11–B3) *showing texture and mineralogy of limestone:* calcite matrix enlarged using SEM in (L11–B2) and minerals identified using XRD in (L11–B3).

Mineralogical and textural evaluation was conducted on 27 selected carbonate rock samples covering the entire study area. The representative and detailed evaluation were performed on two selected carbonate rock samples, L2 and L11, discussed in here. As shown in Fig. 4 [L2–A1 and 2], clear dolomite rhombohedra crystals were found within a calcite matrix and cement, which can be described as dolostones. The X-ray diffraction (XRD) studies also show dolomite as the dominant mineral (Fig. 4 [L2–A3]). The chemical composition of the dolostones was approximately 0.8 wt% SiO_2_, 12 wt% MgO, 41 wt% CaO, and 0.3 wt% Fe_2_O_3_ (Table 3). The limestones, classified as fossiliferous limestone and dominated by calcite mineral, contained foraminifera, calcareous algae, and coral reef fragments. They were the most common materials in the study area (Fig. 4 [L11–B1, 2, and 3]). The chemical composition of the fossiliferous limestone was approximately 0.5 wt% SiO_2_, 0.2 wt% MgO, 55 wt% CaO, and 0.1 wt% Fe_2_O_3_ (Table 3). The wackestone facies—particularly those found in fossiliferous limestone—indicate sedimentary deposition in a shallow marine environment.

**Table 3:** Chemical analyses of the selected limestone.

Samples	wt%								
	SiO_2_	Al_2_O_3_	Fe_2_O_3_	MnO	MgO	CaO	K_2_O	SO_3_	L.O.I
L2	0.77	0.06	0.25	0.02	12.19	40.78	0.11	< 0.01	45.52
L11	0.5	0.07	0.14	0.01	0.22	55.3	0.03	0.01	43.35

## Statistical analyses and discussions

**Figure 5:**
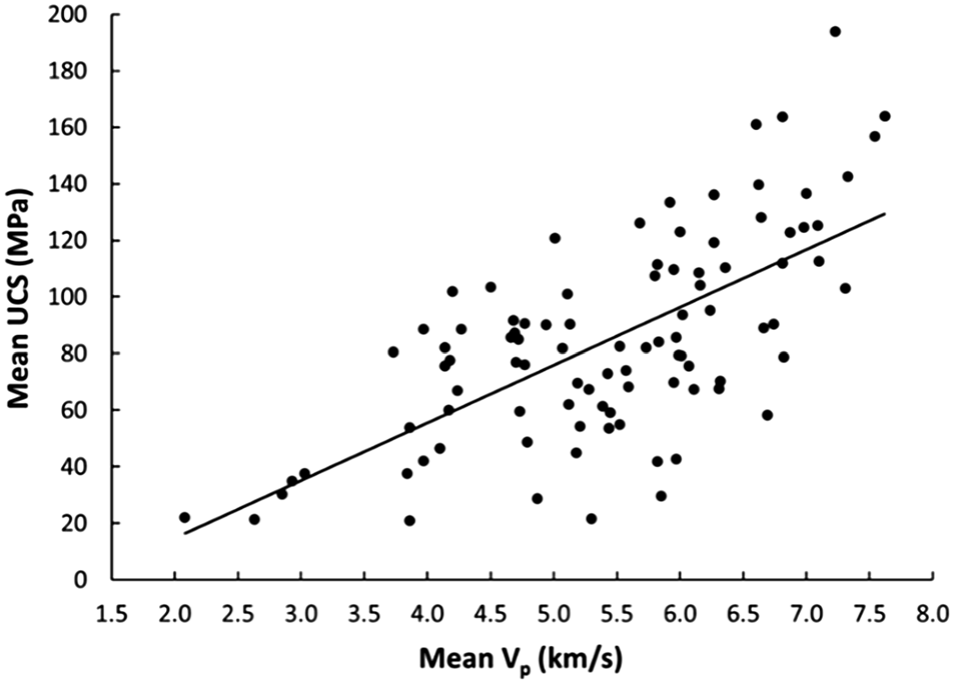
Relationship between the V_p_ and UCS of limestone.

**Figure 6:**
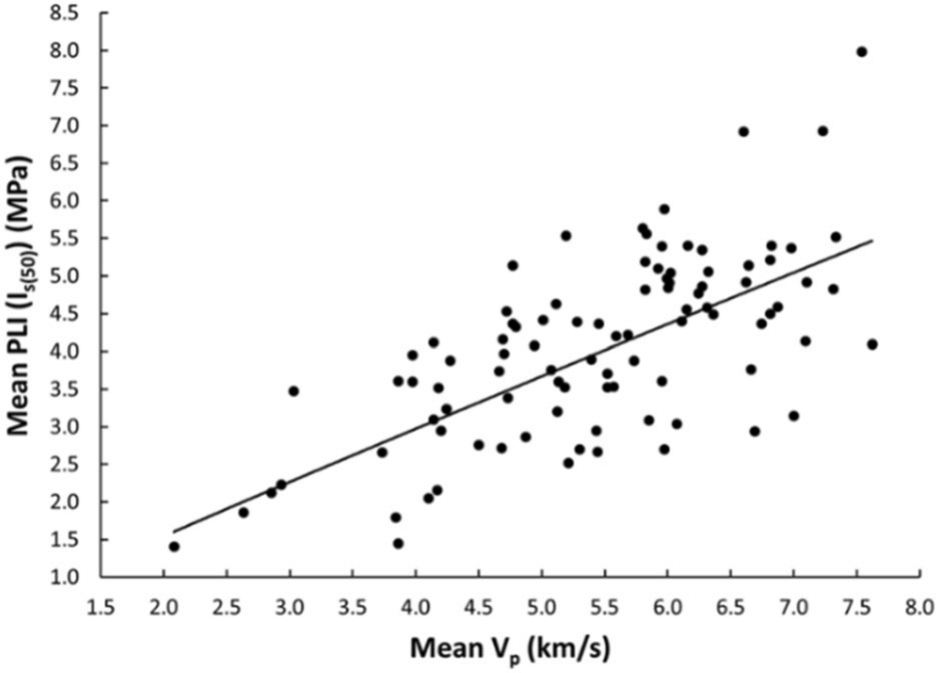
Relationship between the V_p_ and PLI (I_s(50)_) of limestone.

**Figure 7:**
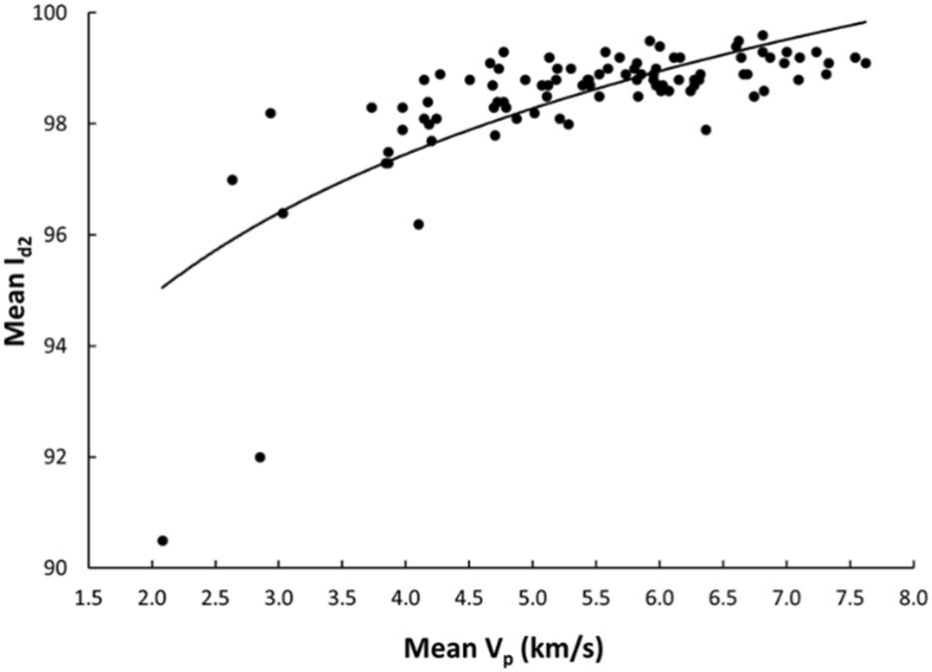
Relationship between the V_p_ and I_d2_ of limestone.

**Figure 8:**
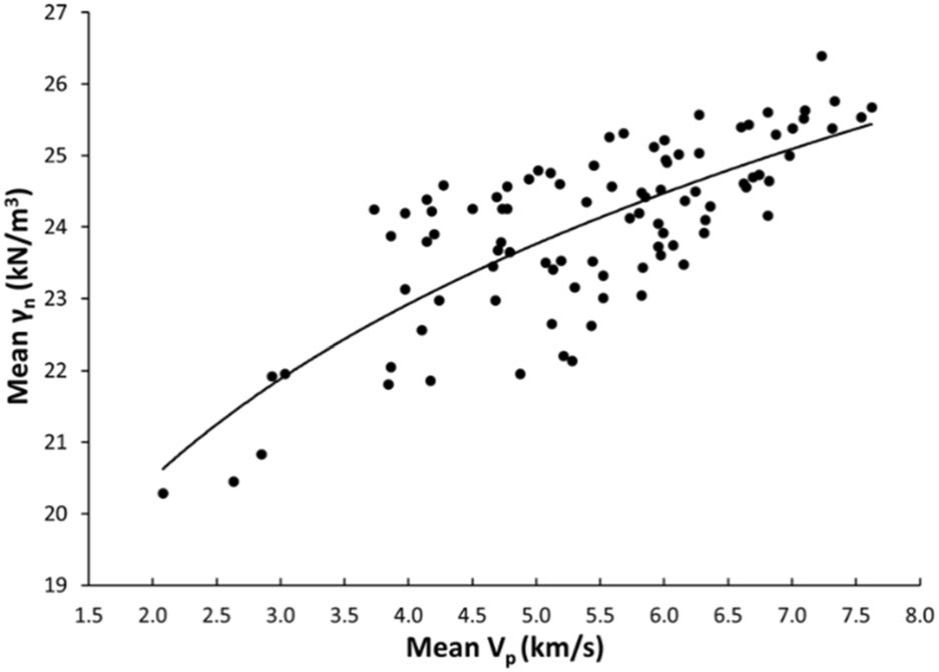
Relationship between the V_p_ and γ_n_ of limestone.

**Figure 9:**
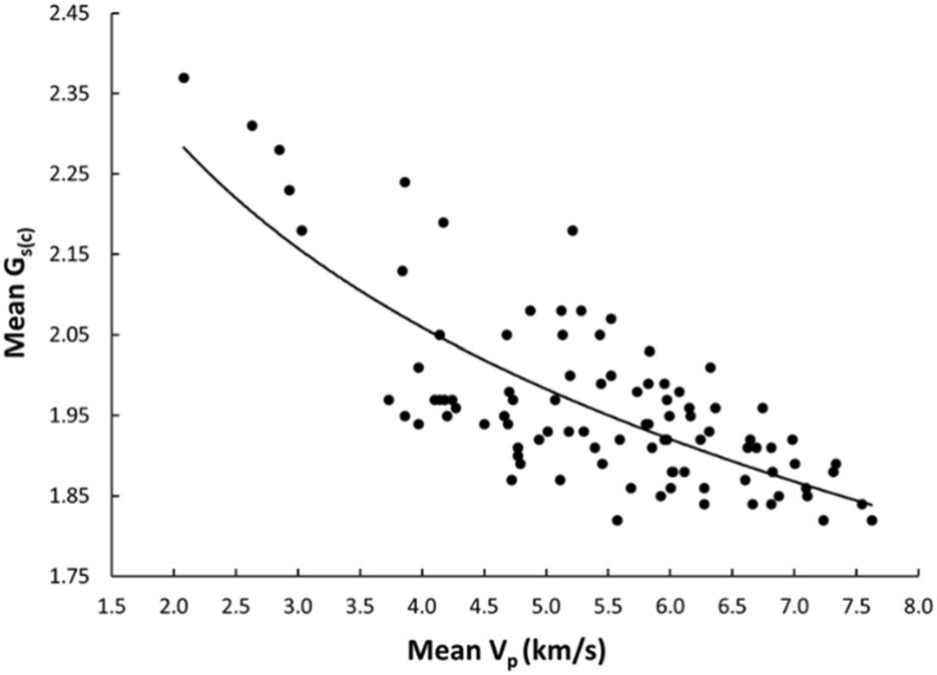
Relationship between the V_p_ and G_s(C)_ of limestone.

**Figure 10:**
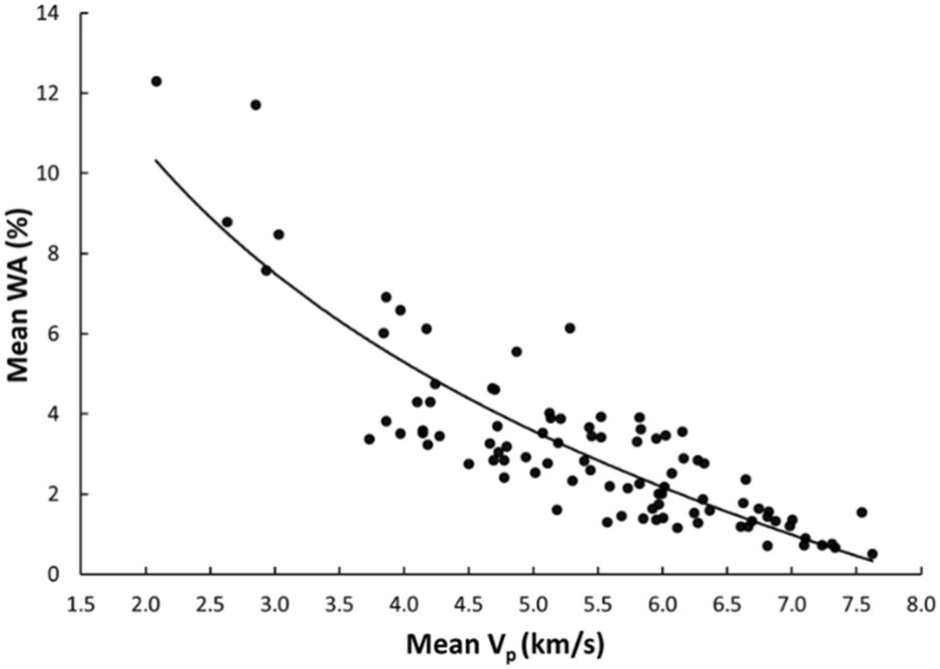
Relationship between the V_p_ and WA of limestone.

**Figure 11:**
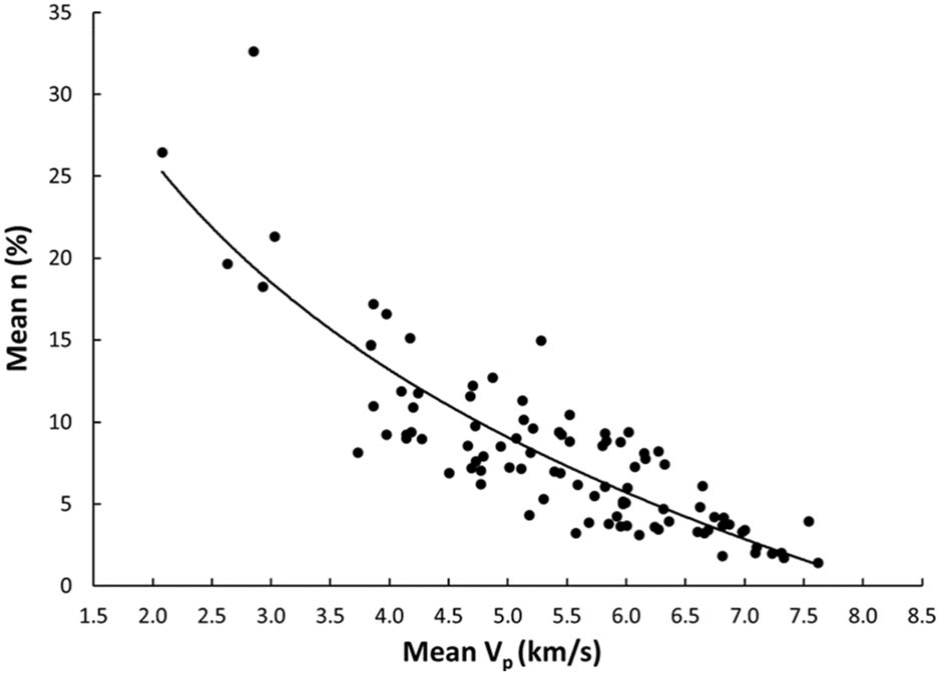
Relationship between the V_p_ and n of limestone.

**Figure 12:**
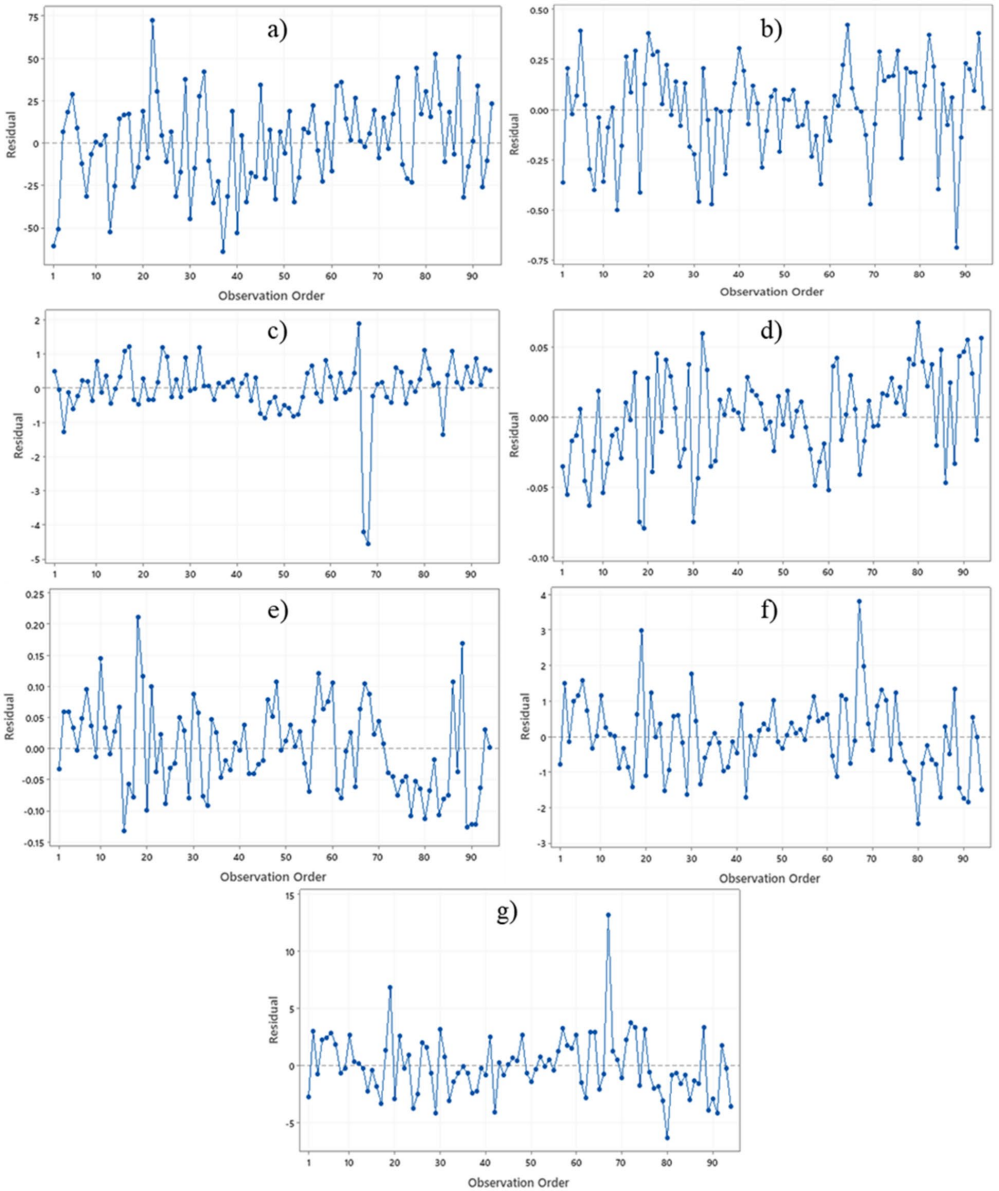
The residual plots of (**a**) UCS versus V_p_, (**b**) PLI(I_s(50)_) versus V_p_, (**c**) I_d2_ versus V_p_, (**d**) γ_n_ versus V_p_, (**e**) G_s(C)_, V_p_, (f) WA versus V_p_ and (**g**) n versus V_p_.

Linear and nonlinear regression analyses are the commonly used and accepted methods for investigating empirical relationships between the mechanical and physical properties of rocks. Numerous researchers have already suggested the empirical relations between the mechanical and physical properties for specific rock types; however, studies only on carbonate/limestone were considered herein (Table 1). The rock type, composition, porosity, water content, and joints have a significant impact on the mechanical and physical properties of rocks. In this study, linear and nonlinear regression models were used to investigate the relationships between some mechanical and physical properties of limestone with respect to V_p_. 95% confidences interval for the parameters were constructed. The correlation coefficient R which measures the strength and direction of the relationship between two variables for each regression mode was computed using the best line fit equation (Figs. 5, 6, 7, 8, 9, 10, 11). Table 4 lists the R-values. Regression analysis revealed a strong relationship between the V_p_ and the PLI, I_d2_, γ_n_, G_s(c)_, WA, and n and a moderate relation between V_p_ and UCS. Furthermore, as far as independence of the residuals is concerned, the residual plots for those models; UCS, PLI(I_s(50)_) I_d2_, γ_n_, G_s(C)_, WA, n and V_p_ are presented in Fig. 12 (a–g). 95% confidence intervals for the true parameters are also given in Table 4.

**Table 4:** Empirical equations between V_p_ and some tested properties of limestone.

Limestone properties	Equations	R-value	*t* tests for the parameter testing			
			t-value	t-critical value	*p*-value < α = 0.05	Confidence intervals
**Mechanical**						
UCS (MPa)	UCS = 20.395V_p_ – 25.968	0.67	8.58	1.662	0.000	(18.03,22.79)
PLI (I_s(50)_) (MPa)	PLI (I_s(50)_) = 0.8023V_p_^0.9448^	0.71	9.54	1.662	0.000	(0.847,1.045)
I_d2_	I_d2_ = 3.6869ln(V_p_) + 92.343	0.72	10.06	1.662	0.000	(3.32,4.05)
**Physical**						
γ_n_ (kN/m^3^)	γ_n_ = 18.328V_p_^0.1614^	0.76	11.37	1.662	0.000	(0.147,0.176)
G_s(C)_	G_**s(c)**_ = –0.342ln(V_p_) + 2.5333	–0.76	–11.1	1.662	0.000	(–0.373, –0.312)
WA (%)	WA = –7.692ln(V_p_) + 15.956	–0.87	–17.07	1.662	0.000	(–7.849, –7.247)
n (%)	n = –18.47ln(V_p_) + 38.8	–0.86	–16.2	1.662	0.000	(–19.62, –17.34)

The validity of the models were tested using Student’s *t* test, and the confidence levels were set at 95% and 0.05 (α = 0.05), respectively (Table 4).

**Figure 13:**
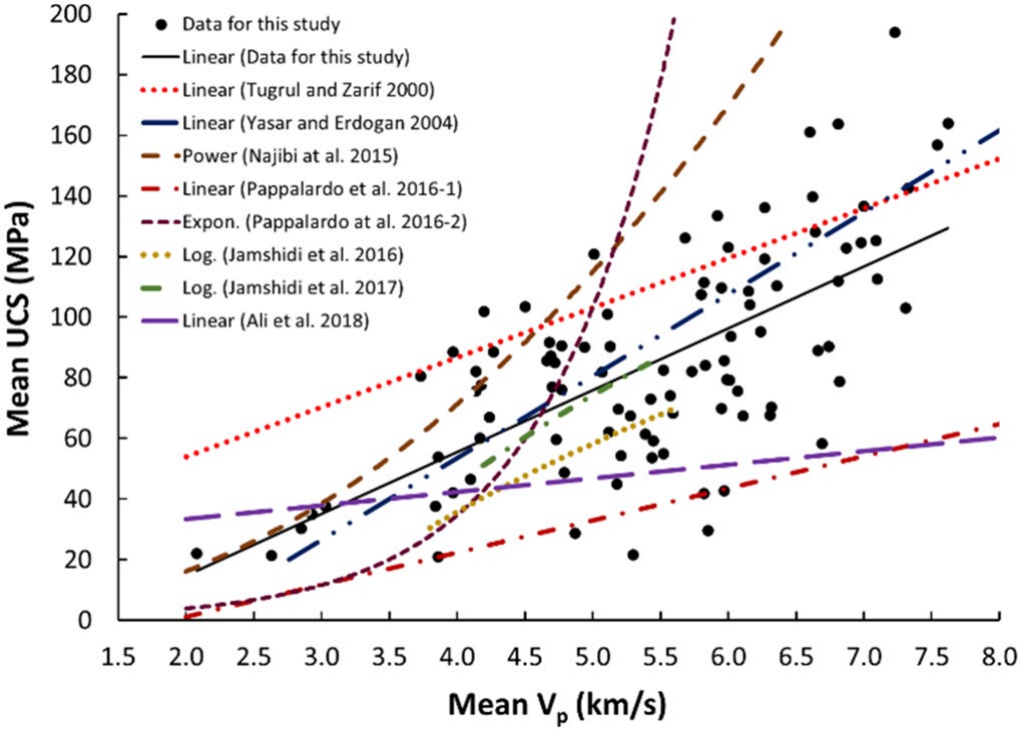
Comparison of results obtained in this study with those obtained in previous studies; V_p_ versus UCS of limestone.

**Figure 14:**
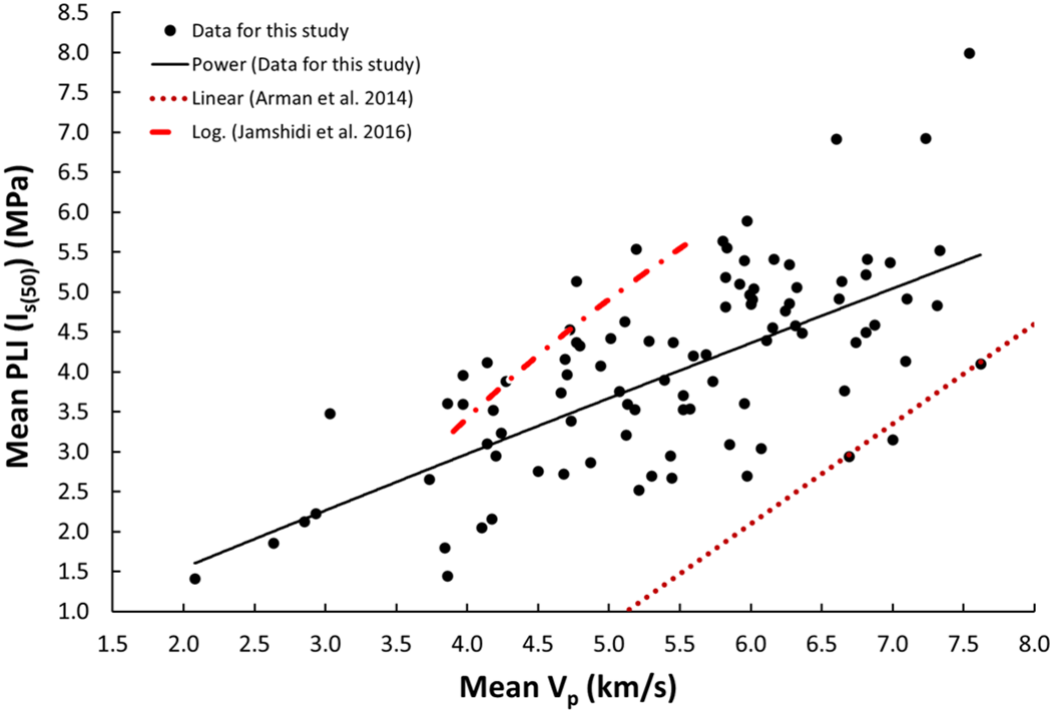
Comparison of results obtained in this study with those obtained in previous studies; V_p_ versus PLI (I_s(50)_) of limestone.

**Figure 15:**
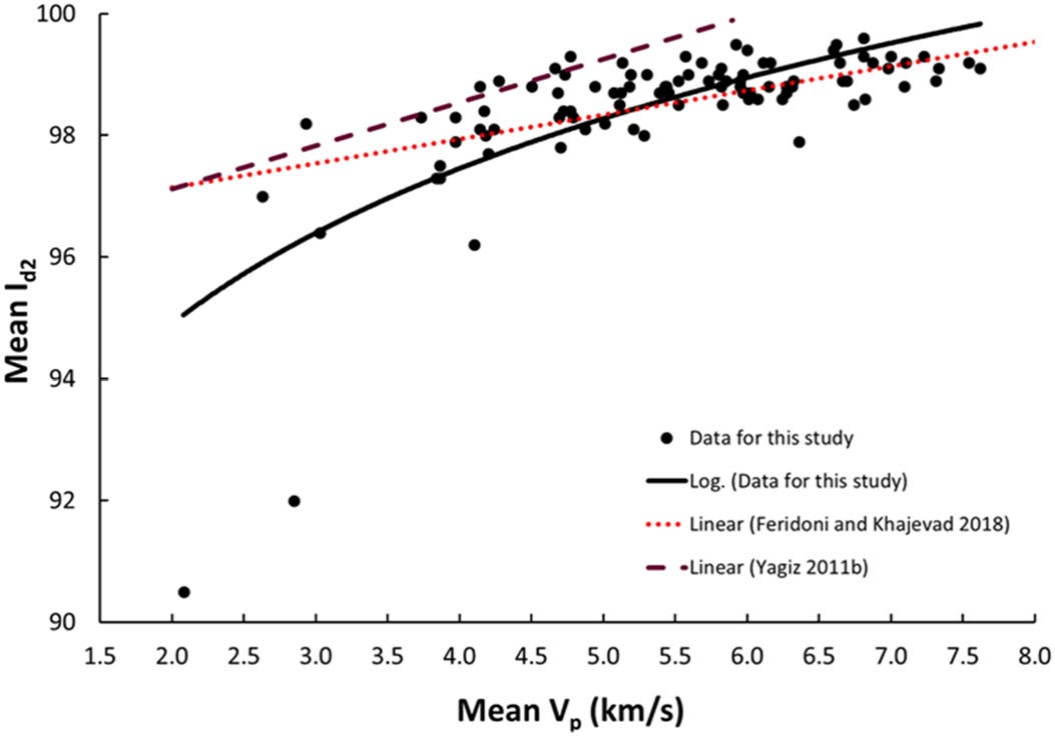
Comparison of results obtained in this study with those obtained in previous studies; V_p_ versus I_d2_ of limestone.

**Figure 16:**
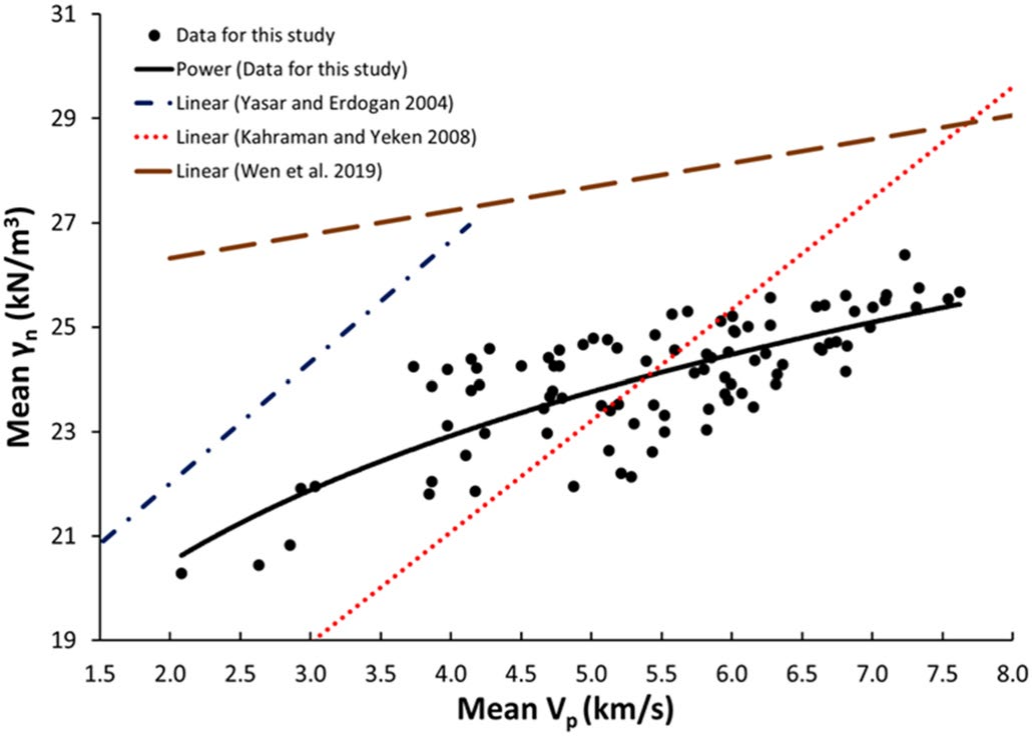
Comparison of results obtained in this study with those obtained in previous studies; V_p_ versus γ_n_ of limestone.

**Figure 17:**
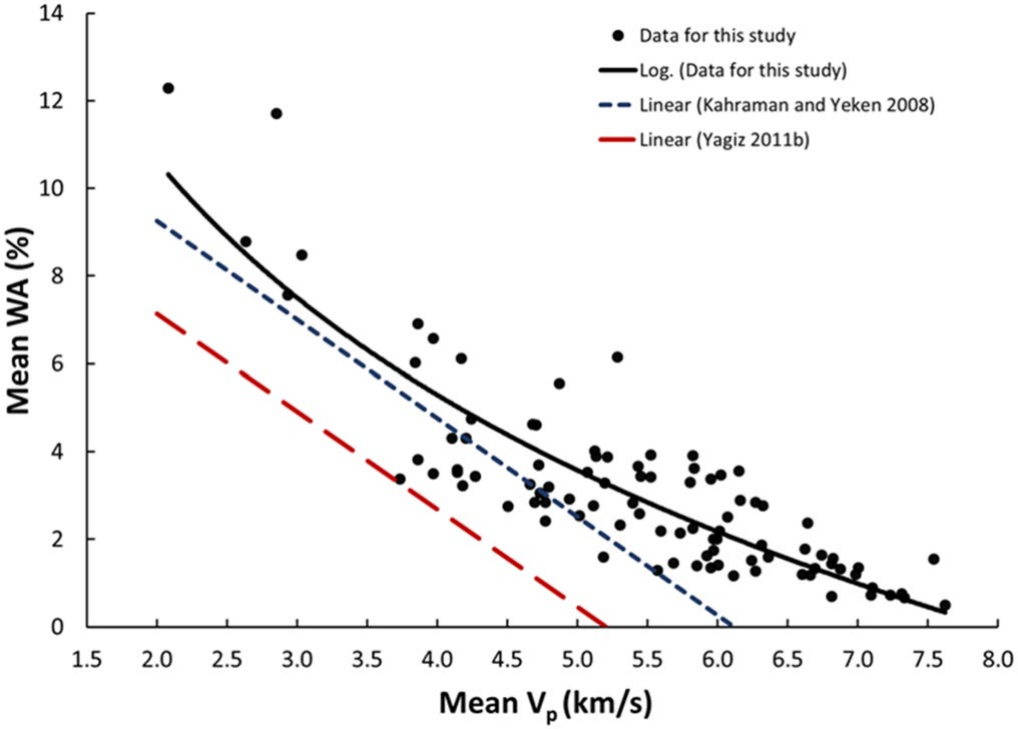
Comparison of results obtained in this study with those obtained in previous studies; V_p_ versus WA of limestone.

**Figure 18:**
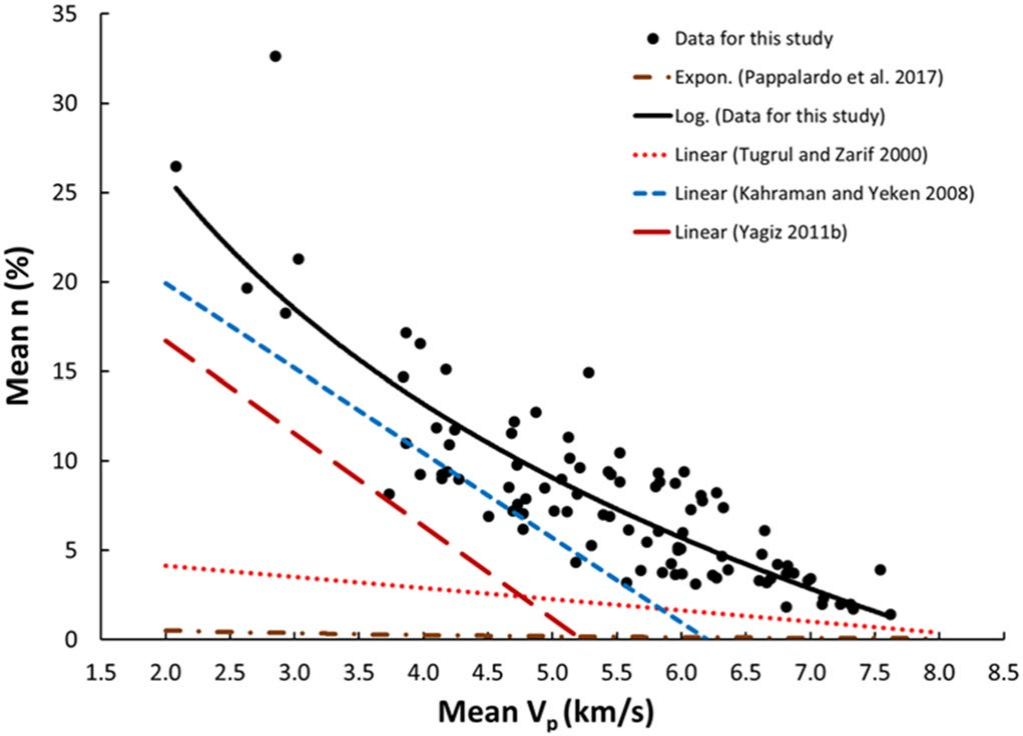
Comparison of results obtained in this study with those obtained in previous studies; V_p_ versus n of limestone.

The derived equations for limestone were compared with the available equations for the same rock types in the literature (Table 1). The relationships between V_p_ and UCS, PLI, I_d2_, γ_n_, G_s(c)_, WA, and n obtained from this study is compared with those of obtained in previous studies (Figs. 13, 14, 15, 16, 17, 18). The R–values, which are considered a good indicator for the strength and direction of the relationship between two variables for each regression model, were computed using the best line fit equations like linear, exponential, power and logarithmic. Those models were chosen based on the empirical distribution of obtained data. As shown in Table 1 and Figs. 13, 14, 15, 16, 17 and 18, the computed equations varied and the regression coefficient (R) ranged from 0.46 to − 0.98. Such variations can be attributed to the origin and other features of rocks, including composition, porosity, and water content.

**Figure 19:**
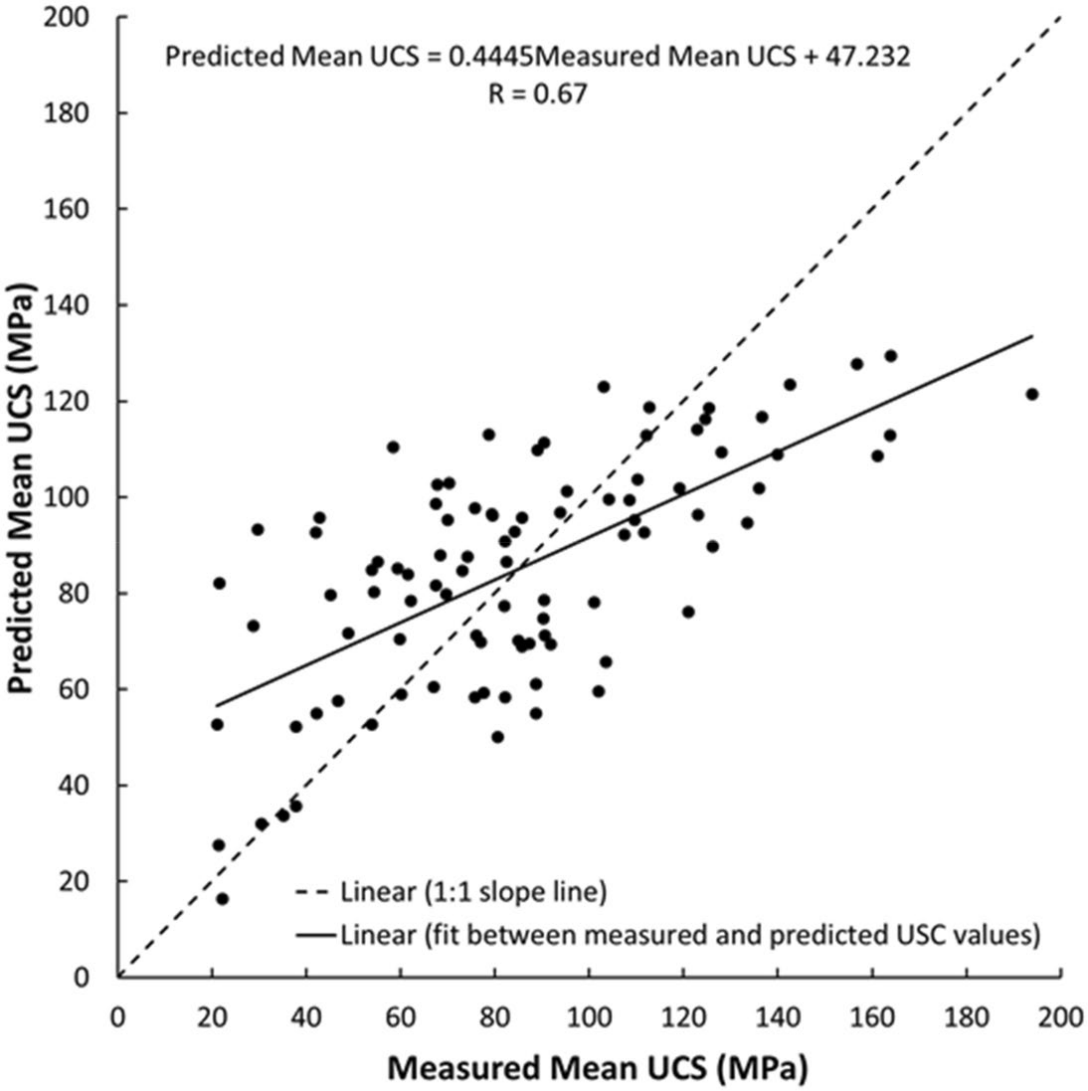
Cross-correlation of the measured and predicted values estimated from V_p_ in terms of UCS values of limestone.

**Figure 20:**
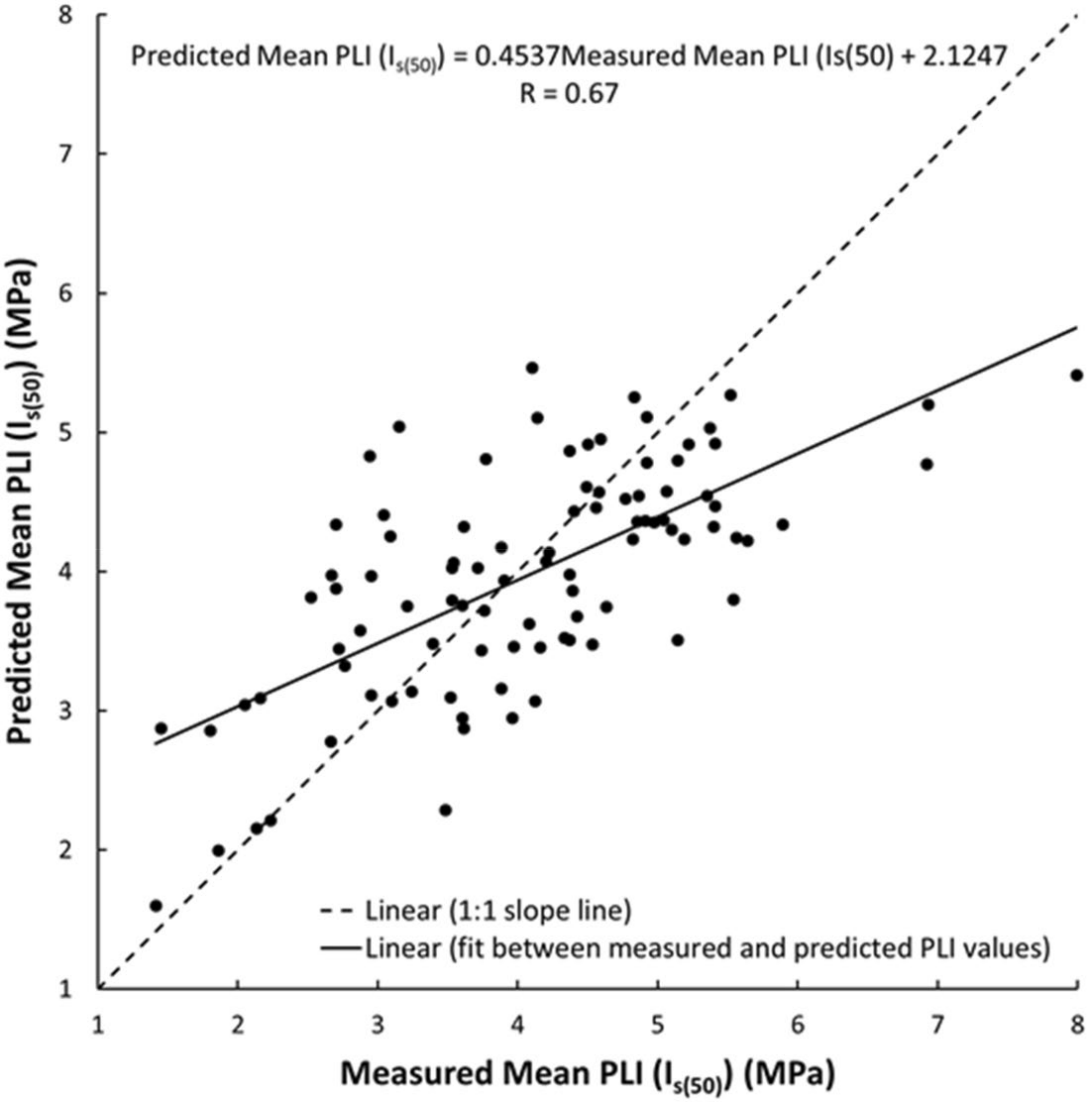
Cross-correlation of the measured and predicted values estimated from V_p_ in terms of PLI (I_s(50)_) values of limestone.

**Figure 21:**
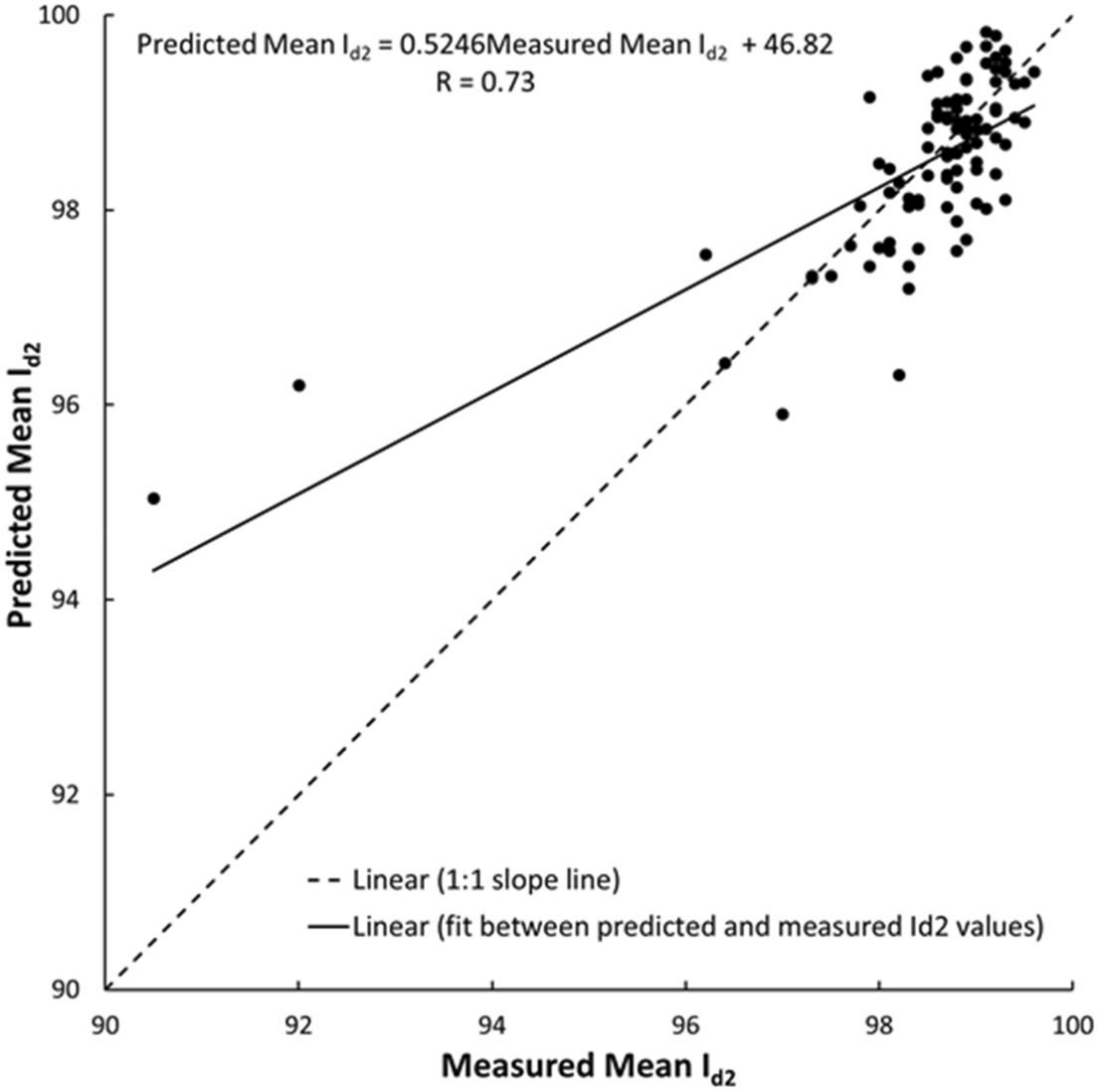
Cross-correlation of the measured and predicted values estimated from V_p_ in terms of I_d2_ values of limestone.

**Figure 22:**
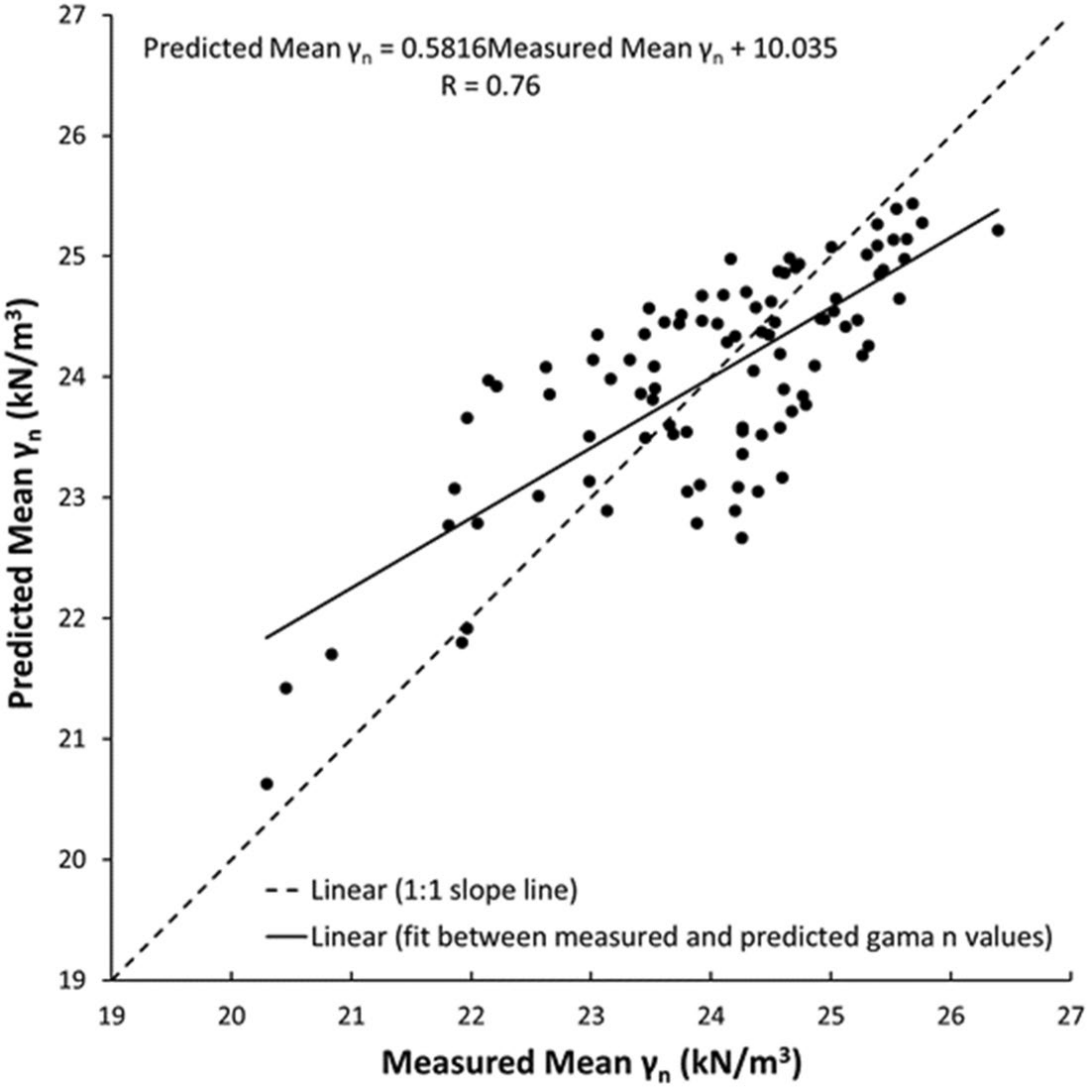
Cross-correlation of the measured and predicted values estimated from V_p_ in terms of γ_n_ values of limestone.

**Figure 23:**
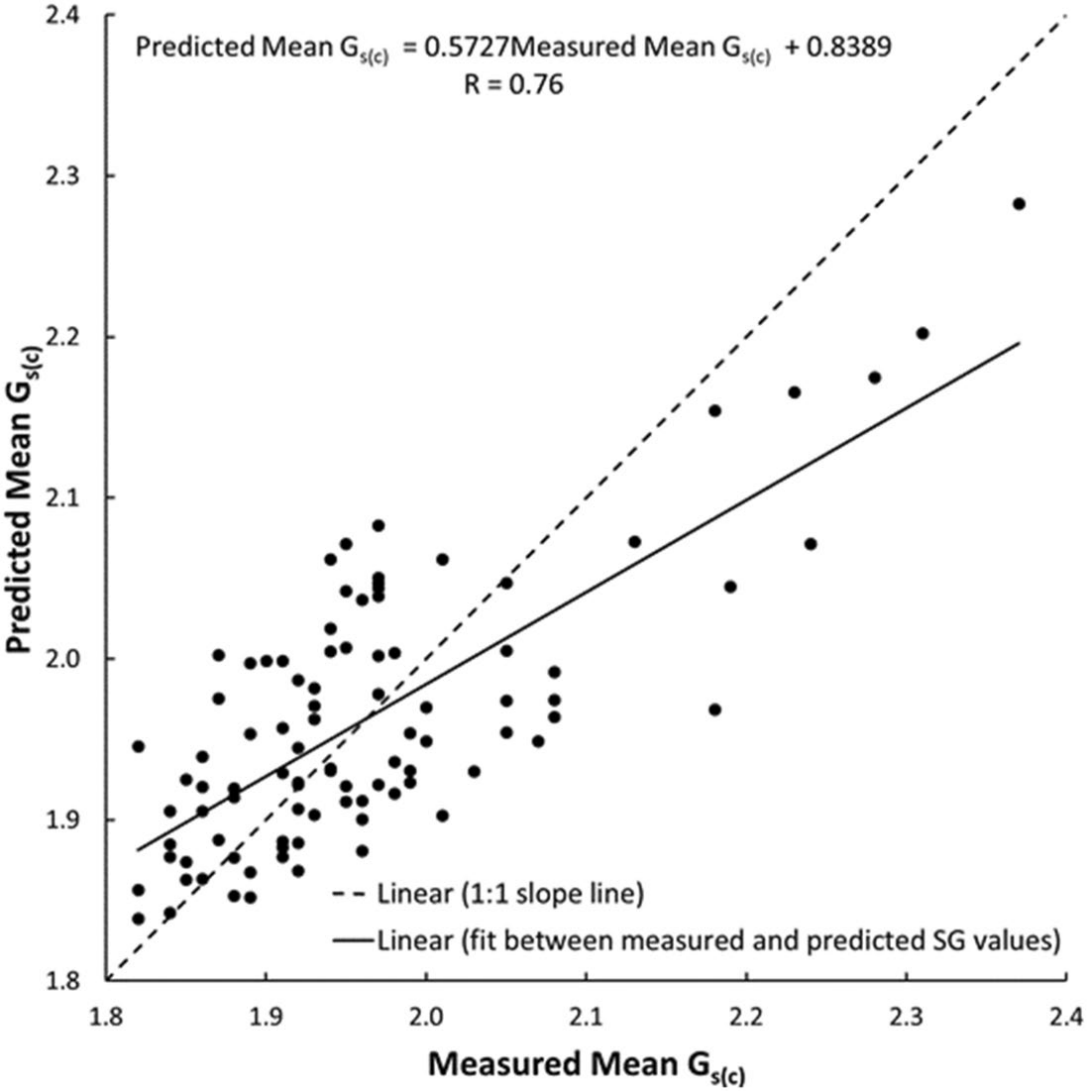
Cross-correlation of the measured and predicted values estimated from V_p_ in terms of G_s(C)_ values of limestone.

**Figure 24:**
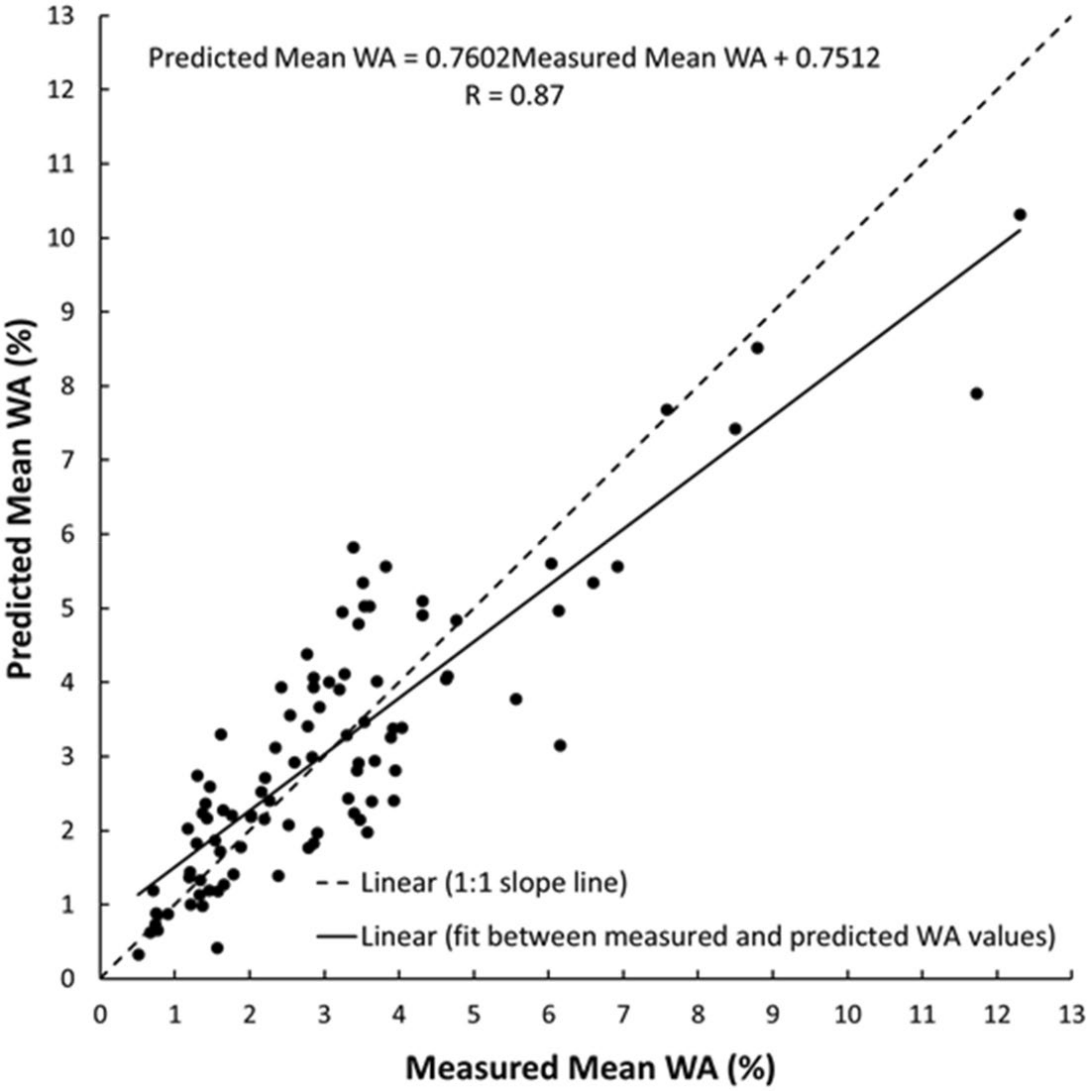
Cross-correlation of the measured and predicted values estimated from V_p_ in terms of WA values of limestone.

**Figure 25:**
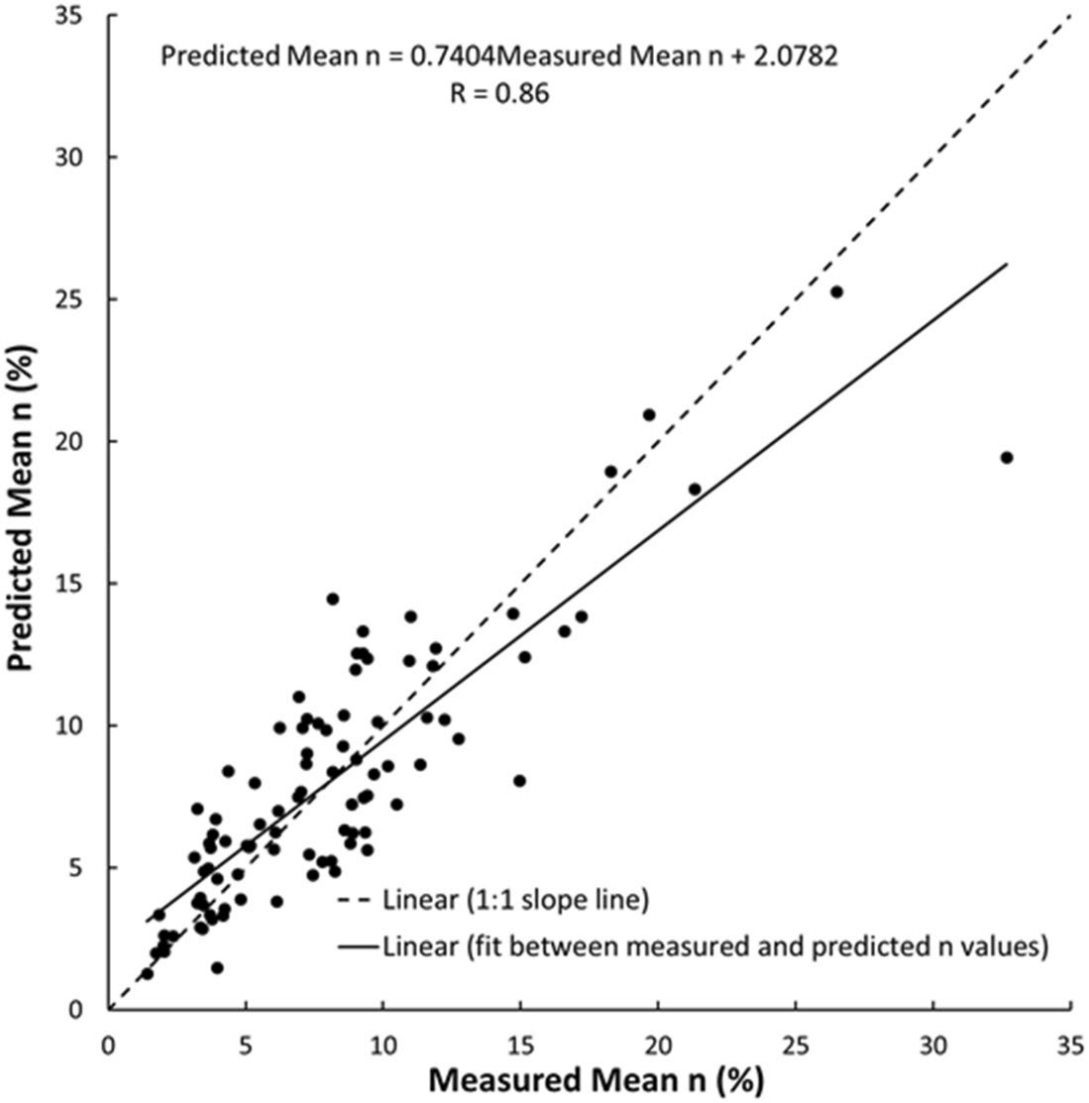
Cross-correlation of the measured and predicted values estimated from V_p_ in terms of n values of limestone.

As shown in Figs. 19, 20, 21, 22, 23, 24 and 25, the predicted and measured values, which are acquired from V_p_, were cross-correlated. The best fit between the measured and predicted values can be evaluated with the 1:1 slope line that indicates a perfect correlation; the accuracy level of the measured values declined with an increased deviation from the 1:1 slope line. This study proves the reliability of estimating the mechanical and physical properties of limestone from V_p_ values.

## Conclusions

An intensive experimental program—over 1750 tests on limestone samples collected from seven different locations along the study—were conducted using several standard testing methods. Regression analyses, both linear and nonlinear, were used to generate empirical correlations between V_p_ and the mechanical and physical features of limestone, i.e., UCS, PLI, I_d2_, γ_n_, G_s(c)_, WA, and n. Based on the test results, the following conclusions were accomplished:

1. The statistical analyses indicate significant correlations between V_p_ and UCS, PLI, I_d2_, γ_n_, G_s(c)_, WA, and n. Thus, a V_p_ test—a simple, fast, economical, and nondestructive method for characterizing rock—can be used to predict the UCS, PLI, I_d2_, γ_n_, G_s(c)_, WA, and n of limestone.
2. The correlation of coefficient, R, between UCS, PLI, I_d2_ and V_p_ range 0.67–0.72.
3. The WA, n, γ_n_, and G_s(c)_ of limestone provide the best correlation with V_p_ (R =  − 0.87, − 0.86, 0.76, and − 0.76, respectively).
4. For all cases, the calculated t-test statistics were highly significant which confirm the UCS, PLI, I_d2_, γ_n_, G_s(c)_, WA, and n of limestone in the study area can be reliably estimated using the proposed correlation equations.

While the results of this study may have wide common usage in engineering applications, the provided equations apply only for the specified rock. Further research is necessary to apply these results to other rock types.

### Statistical analysis

Descriptive statistics of the data is presented in Table 2. Since the sample size is large (n = 94), the intervals within one standard deviation of the means are calculated using the empirical rule to check the data spread. The Statistical package, Minitab, is used to investigate the empirical correlations between various parameters through linear and nonlinear regression analyses. To test the validities of the regression models, the Student’s* t* tests statistics, critical values, and the p-values of the variable relationships, as well as the 95% confidence intervals of the true parameters are summarized in Table 3. According to the central limit theorem, there is no need to verify normality for large samples (n >  = 30) since the sample mean is approximately normally distributed.
